# A novel approach to detecting microduplication in split hand/foot malformation type 3 at the single-cell level: SHFM as a case study

**DOI:** 10.1186/s13023-024-03386-5

**Published:** 2024-10-31

**Authors:** Yaqian Wang, Yang Li, Lidong Zeng, Wenbo Li, Xin Dong, Jia Guo, Xiangrui Meng, Jiacheng Lu, Jiawei Xu

**Affiliations:** 1 The First Affiliated Hospital, Tianjian Laboratory of Advanced Biomedical Sciences, Zhengzhou University and Institute of Reproductive Health, Henan Academy of Innovations in Medical Science, Zhengzhou, China; 2 NHC Key Laboratory of Birth Defects Prevention, Zhengzhou, China; 3 Shenzhen GeneMind Biosciences Co., Ltd, Shenzhen, China

**Keywords:** Split hand/foot malformation, Microduplication, Whole-genome amplification, Next-generation sequencing, Karyomapping

## Abstract

**Background:**

Split hand/foot malformation (SHFM) is a congenital limb deficiency characterized by missing or shortened central digits. Several gene loci have been associated with SHFM. Identifying microduplications at the single-cell level is challenging in clinical practice, and traditional detection methods may lead to misdiagnoses in embryos and pregnant women.

**Results:**

In this research, we utilized a low cell count and whole-genome amplification products to employ single nucleotide polymorphism arrays, next-generation sequencing, and third-generation sequencing methods to detect copy number variants of microduplications in a SHFM3 case with limited DNA. Additionally, Karyomapping and combined linkage analysis were conducted to validate the results.

**Conclusions:**

This study establishes a new strategy for identifying microduplications or microdeletions at the single-cell level in clinical preimplantation genetic testing, enhancing the efficiency and accuracy of diagnosing microduplication or microdeletion diseases during IVF-PGT and prenatal diagnosis.

## Background

Split hand/foot malformation (SHFM) is a distal limb malformation characterized by missing or shortened central digits, often accompanied by fusion of the remaining digits and median clefts of the hands and/or feet [1]. The estimated incidence of SHFM in China is 1.64/1000 [2, 3]. SHFM exhibits phenotypic variability among individuals, ranging from syndactyly and oligodactyly to the most severe form, monodactyl. Limb bud formation relies on signaling molecules produced by three specific cell populations: apical ectodermal ridge (AER), progression zone (PZ), and polarization active zone (ZPA) [4]. The primary pathogenesis of SHFM is the failure to maintain median apical ectodermal signal transduction [5]. In addition to genetic factors, environmental factors, including apoptosis in AER, also contribute to SHFM [6].

Typically, SHFM follows an autosomal dominant inheritance pattern, but it can also manifest as autosomal recessive inheritance or X-linked. Hand-foot cleft malformation type 1 (SHFM1) results from a chromosomal rearrangement in the 7q21.3-q22.1 region [7, 8] and is primarily autosomal dominant, potentially associated with isolated or syndromic limb deformities [9]. This complex syndrome is linked to dysfunction in the *DSS1, DLX5,* and *DLX6* genes [10, 11]. Hand-foot malformation type 2 (SHFM2) occurs at the Xq26 locus and exhibits X-linked [12, 13]. The SHFM3 is located at chromosome 10q24, making it the most prevalent type of SHFM in humans [14]. Hand-foot cleft malformation type 4 (SHFM4) is associated with the gene located at 3q27, featuring mutations in the *TP63* (also known as *P63*) gene and an autosomal dominant inheritance pattern [15, 16]. Hand-foot cleft malformation type 5 (SHFM5) is located at 2q31 and may result from autosomal dominant inheritance due to HOXD cluster deletion [17, 18]. Hand-foot cleft malformation type 6 (SHFM6), situated at 12q13, is an autosomal recessive inherited disease caused by a homozygous mutation of the *WNT10B* gene [19, 20]. Split hand/foot malformation with long bone deficiency (SHFLD) is defined as a complex form of split had/foot malformation involving the tibia and fibula, which is most commonly associated with duplication of the 17p13.3 locus [21].

Genetic variations and chromosomal rearrangements are well-established as the primary drivers of genomic diseases, impacting fertility and embryo development. Prenatal diagnosis and preimplantation genetic testing offer effective strategies to prevent the transmission of pathogenic variations to offspring. These include preimplantation genetic testing for aneuploidy (PGT-A), monogenic disorders (PGT-M), and structural rearrangements (PGT-SR) [22]. As integral components of assisted reproduction technology (ART), these methods have been collectively employed to reduce fetal genetic risk and enhance pregnancy rates. However, PGT platforms involve numerous workflow steps, leading to high costs and prolonged operation times. These limitations, stemming from the scarcity of available cells and the extended process, restrict their widespread application [23]. Although PGT-A has found extensive use in detecting embryo copy number variants (CNVs), its typical resolution remains around 5–10 Mb [24]. Advances in whole-genome amplification (WGA) and next-generation sequencing (NGS) technologies have only marginally improved this resolution to approximately 1 Mb [25], making it challenging to achieve resolutions below 1 Mb for prenatal diagnosis/screening and PGT-A with limited biopsy samples. Recent PGT-M efforts successfully addressed a 108 kb chr14q32 microdeletion in a family with Kagami-Ogata and Temple syndromes, suggesting the potential utility of PGT-M in preventing the transmission of rare genomic diseases [26]. However, no established recommendations or guidelines exist for PGT-M concerning small CNVs.

Furthermore, the boundaries of PGT for small CNVs remain unclear, given the highly heterogeneous symptoms observed in individuals with such CNVs [27]. Diagnosing small CNVs at the single-cell level is a challenging task. Therefore, our objective is to devise comprehensive strategies for the detection of small CNVs using only a limited number of cells or DNA samples. This initiative aims to establish a novel comprehensive PGT/limited DNA strategy for identifying micro-variations in a clinical setting, ultimately enhancing diagnostic accuracy and preventing the transmission of genomic diseases to offspring.

## Methods

### Ethics statement

This study received approval from the Institutional Review Board of The First Affiliated Hospital of Zhengzhou University (2022-KY-1347). Standard protocols were adhered to during sample collection.

### DNA extraction

The genomic DNA was extracted using TIAGEN (DP304) according to the manual. DNA quality was evaluated by NanoDrop™ spectrophotometer (Thermo Fisher Scientific, Inc.), and quantified using the Qubit® 3.0 fluorometer (Thermo Fisher Scientific, Inc.), then the DNA stored at − 80 °C before use.

### Limited DNA whole genome amplification

Because of the small amount of genetic material, genomic analyses of a single or a few cells require whole-genome amplification [28]. The amplification of limited DNA samples was achieved through multiple displacement amplification (MDA) using the QIAGEN REPLI-g Single Cell Kit (Germany). Additionally, the multiple annealing and looping-based amplification cycles (MALBAC) method from Yikon was employed to generate a substantial amount of DNA. All procedures strictly followed the provided instructions.

### Cnvs detection method based on single nucleotide polymorphism (SNP) assay

The SNP array based CNV detection method involves hybridizing the test sample with a chip probe, enabling the determination of copy numbers at each site by comparing signal intensities across different samples. This is accomplished by examining the ratios of two allelic fluorescence signals, denoted as A and B. The Log R Ratio (LRR) is calculated as LRR = Log2(R observed/R expected), where R is the sum of the intensities of the A and B allelic fluorescence signals. “Observed” corresponds to values detected in the experimental samples, while “expected” signifies the values fitted by an algorithm, representing measurements from normal samples. LRR serves as an indicator of copy number change relative to normal samples, with LRR = 0 signifying normal copy numbers, LRR > 0 indicating an increase in copy numbers, and LRR < 0 suggesting a decrease in copy numbers. Furthermore, the B Allelic Frequency (BAF) measures the ratio of two allelic signal intensities (BAF = normalized measure of relative signal intensity (B/A)). Notably, despite there being only three possible typing results, BAF values can fluctuate within the range of 0–1, reflecting a certain degree of disturbance in fluorescence signal intensity. It is essential to emphasize that the fluorescence signal intensity is normalized. Based on statistical analysis of BAF and LRR, the algorithm can determine the copy number of the corresponding genomic region.

### Cnvs detection method based on high throughput sequencing

Genome-wide CNVs detection was carried out on patient DNA samples, with the experimental results analyzed using Genome Studio. The identified CNVs were compared to the internationally available normal DGV (http://projects.tcag.ca/variation) and queried in phenotype databases, including c (http://decipher.sanger.ac.uk/information), ClinVar (http://www.ncbi.nlm.nih.gov/clinvar), OMIM database (http://www.ncbi.nlm.nih.gov/omim), and the PUBMED database to assess the presence of known disease-associated CNVs. Variants were analyzed following the Standards and Guidelines for the Interpretation of Sequence Variants established by the American College of Medical Genetics and Genomics [29]. Mutation nomenclature adhered to the Human Genome Variation Society (HGVS) standards (http://www.hgvs.org/mutnomen/) [30].

### The karyomapping method detect the cnvs

The Karyomapping method is employed for preimplantation genetic diagnostics. It operates by comparing DNA samples from embryos with those of parents and close relatives and constructing a genealogy map to screen for genetic disease genes, similar to identifying unique genetic markers on chromosomes, similar to fingerprints. In this experiment, DNA samples from fetal tissue, trace DNA-amplified samples, and peripheral blood DNA from three family members were subjected to analysis using the Karyomap chip. Amplified DNA was scanned on the HumanKaryomap-12 Bead Chips (Illumina) platform and analyzed with Blue-Fuse Multi software (Illumina, San Diego, CA, USA) to determine nuclear localization arrays.

### Single-molecule gene sequence method detect cnvs

#### Library construction and sequencing

The method for detecting CNVs at the single-molecule gene sequence level is explicated herein. The process commences with library construction and sequencing, wherein DNA fragmentation is undertaken using the FEA Enzyme mix (Vazyme) per the manufacturer’s guidelines. Subsequently, the SMS library is created. The SMS library protocol includes the ensuing stages: (1) end repair and A tailing, (2) adapter ligation, and (3) beads cleanup. Following DNA concentration quantification, the libraries are dissolved, denatured, and subsequently loaded into the GenoCare 1600 sequencer. Diverging from previous methodologies involving poly-A tailing and blocking, we have devised a double-stranded DNA adaptor possessing overhangs at both 3′ termini. These extensive (> 20 base pairs) DNA overhangs are hybridized to the probes situated on sequencing flow cells. The adaptor also includes additional bases at its 3′ extremity to forestall extension from the library strand’s 3′ end. Contrarily, the new flow cell, as opposed to its precursor, incorporates probes constituted by a DNA oligo that complements the lengthy overhangs of the adapter. In this context, not only DNA fragments with both ends ligated with adapters but also those with a single end ligated can serve as efficacious sequencing templates within the library. This novel library structure, in contrast to the poly-T adaptor library, serves to stabilize the hybridization between the surface probe and the adapter. Furthermore, it enables direct sequencing from the surface probe’s 3′ end, obviating the need for the fill-lock step requisite in poly-T-to-poly-A capture. The GenoCare 1600 sequencer (Shenzhen GeneMind Biosciences Co., Ltd.) includes 16 physically segregated lanes, with each sample library allocated to a distinct lane.

#### Data analysis

The SNP array and Karyomapping Chip data were analyzed in accordance with the provided instructions. Next-generation sequencing data underwent analysis utilizing a custom-developed pipeline. For single molecular sequencing, SMS data mapping was facilitated through the utilization of the in-house developed software known as “directAlignment”. Initially, reads with lengths exceeding 28 base pairs were subjected to global alignment against the reference genome (hg19) employing a prebuilt hash table. It is pertinent to note that multiple mapping sites were often identified for the majority of reads during this phase. Subsequently, candidate mapping sites were subjected to filtration employing a rapid bit-vector algorithm designed for approximate string matching, founded on dynamic programming principles. Following this, the remaining candidate mapping sites underwent further alignment with the reads utilizing the Smith-Waterman local alignment algorithm. This process typically resulted in the retention of only one or a limited number of optimal mapping sites for each read. Thereafter, a stringent filtering regime was applied, including both a maximum mapping error rate constraint and a minimum mapping read length requirement. Reads that failed to meet the stipulated maximum error tolerance were methodically truncated from both ends, base by base, until compliance with the error tolerance was achieved or their length dwindled below the minimum length criterion. Following this rigorous filtering procedure, it was observed that about 5 M unique reads per sample were successfully mapped, with an average length of about 36 bp, utilizing the GenoCare 1600 platform. Subsequent to mapping, the genome was partitioned into 20-kb non-overlapping bins, and raw counts were obtained for each bin. Notably, regions characterized by high variability, extensive repeat regions, and regions located within the telomeric and centromeric regions were excluded from further analysis. It is imperative to mention that GC correction was meticulously implemented through the following steps: the GC content of each bin was computed, and the theoretical read counts corresponding to different GC levels were predicted via locally weighted scatter plot smoothing (LOESS) regression $GC\_loess_{i}$. Subsequently, the GC-corrected read counts (RC_GC_) for each bin were computed as the product of the raw read counts (RC_raw_) and a correlation factor (F). The latter was derived from the median counts of all the bins ($M_{{{\text{global}}}}$) and the LOESS fit predicted value, as outlined below:

$$W_{{\text{i}}} = GC\_loess_{i} /M_{global}$$

The map ability was executed using a comparable methodology. The establishment of the normal reference set entailed the inclusion of data from 100 healthy individuals, including both males (46, XY) and females (46, XX). To identify copy number regions, the circular binary segmentation algorithm, a component of the DNAcopy package in R (version 1.36.0), was proficiently employed.

## Results

### Clinical descriptions of the SHFM family

**Fig. 1:**
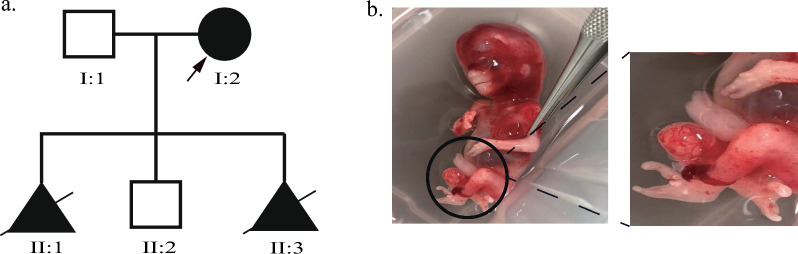
Family pedigree and clinical characteration. Pedigree of the family. I2 is the proband. **a**, **b** Photos of the hands and the feet of the fetus

The proband (I:2) exhibits the characteristic presentation of median division malformed clefts in both hands and feet, resulting in a diagnosis of split hand and feet malformation based on the patient’s clinical manifestations. Notably, the proband’s husband (I:1) demonstrates no clinical abnormalities. As depicted in the pedigree chart (Fig. 1a), the family manifests a hereditary genetic trait indicative of autosomal dominant inheritance, which suggests the likelihood of occurrence in each successive generation. Approximately 3 years ago, the couple experienced a pregnancy resulting in a child with hand-foot deformity (II:1). Following medical advice, they opted for induced labor. Subsequently, the couple welcomed the birth of a healthy son (II:2).

Approximately 4 months ago, the patient became pregnant once again. Ultrasound assessment indicated an intrauterine pregnancy with a single live fetus at a gestational age of 12 weeks and 4 days. Fetal NT measurements fell within the normal range. Further ultrasound observations revealed specific parameters, including a head and hip diameter (CRL) of 60 mm, a double parapedal diameter (BPD) of 21 mm, a heart rate of 151 beats per minute, and a normal cranial appearance with no evidence of anencephaly, open brain deformity, or severe sternoabdominal wall defects. The placenta was situated on the posterior wall with a thickness of 12 mm, and the maximum depth of amniotic fluid reached 35 mm. Notably, the inner cervix was closed. However, the development of both hands and feet in the fetus (II:3) exhibited anomalies characterized by a median laceration. In light of the family’s medical history, the couple made the decision to terminate the pregnancy at 13 weeks (Fig. 1b).

### Chromosome copy number variations screen of the family

**Fig. 2:**
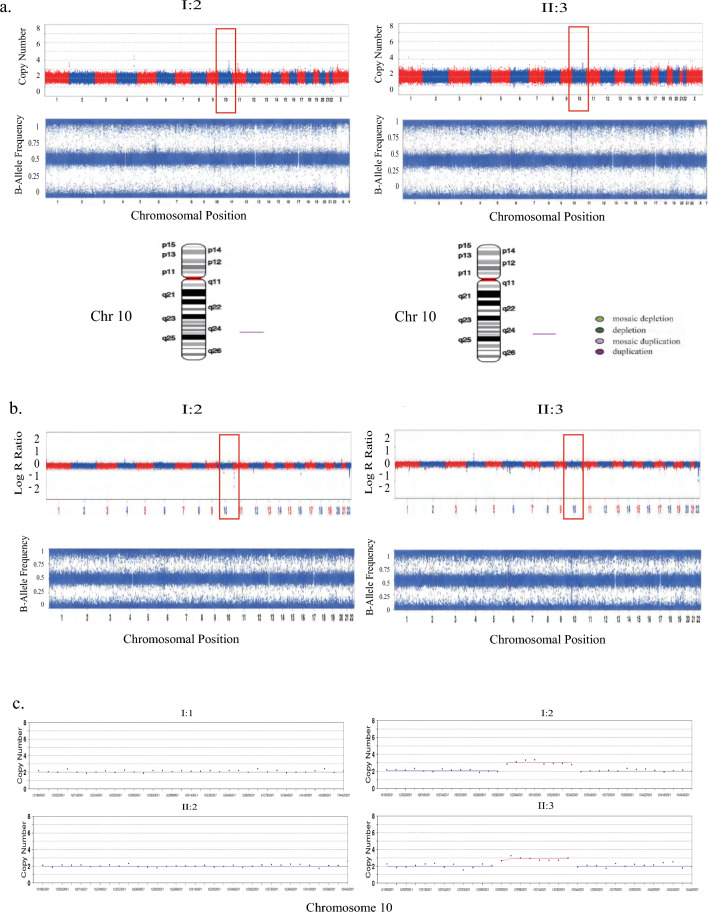
Copy number variation detection in the SHFM3 family using both SNP array and NGS. **a** The result of copy number repeats of low coverage NGS. The proband (left) and the fetus (right). **b** The result of copy number repeats of SNP assay. The proband (left) and the fetus (right). All suggest that the proband and her fetus have the 0.6 Mb microduplication at chromosome 10. **c** The display of CNVs at chr10

Subsequently, the family underwent chromosome copy number variation (CNV) screening. Traditionally, a combination of next-generation sequencing technology and SNP assay was employed for CNV detection, with a focus on chromosome aneuploidy and the presence of fragments exceeding 100 Kb in size. In this specific pedigree, standard DNA samples were subjected to CNV analysis using next-generation sequencing. The results indicated that the husband and their son exhibited no chromosome aneuploidy or CNV at loci related to SHFM. Conversely, both the proband (I:2) and the fetus displayed a microduplication involving chromosome 10 (Fig. 2a). Further validation through SNP array analysis yielded consistent findings (Fig. 2b). In-depth analysis via next-generation sequencing unveiled approximately 0.62 Mb duplications in the q24.31q24.32 region of chromosome 10 for the proband (seq [GRCh37]dup(10)(q24.31q24.32) chr10:g.102860001_103480000dup). Chromosome analysis of the aborted fetal tissue (II:3) demonstrated a similar 0.64 Mb copy number repeat in the q24.31q24.32 region of chromosome 10 (seq [GRCh37]dup(10)(q24.31q24.32) chr10:g.102820001_103460000dup) (Fig. 2c). Importantly, a comprehensive query revealed the presence of several consecutive gene repeats within this genomic region, including *LBX1, BTRC, POLL*, and *DPCD.* These genes significantly overlap with the core pathogenic region associated with congenital hand and foot cleft malformation, as documented in the OMIM database and relevant literature [5]. The evaluation of pathogenicity was conducted in accordance with the guidelines set forth by the American Society of Medical Genetics and Genomics (ACMG) [31], thereby suggesting that the observed variation was likely pathogenetic (LP) (Table 1). Subsequent scrutiny of the DECIPHER database revealed potential clinical manifestations among individuals with duplications in close proximity to this locus, notably hand-foot cleft deformity and the presence of multiple fingers anterior to the axis (DECIPHER ID: 360984, 368251, 368247). No CNV report was discerned within the general population database known as DGV. In summary, through extensive examination of cells utilizing NGS and SNP assays, it was ascertained that the overlap of pathogenic sites between the proband and the fetus included a span of 0.62 Mb, thus indicating a likelihood of pathogeny. Furthermore, no mutations were detected within the healthy son and the husband of the proband.

**Table 1:** Pathogenicity of the family is assessed according to ACMG guidelines

ID	Variation information	Variation type	Length (Mb)	ACMG
I:2	seq[GRCh37]dup(10)(q24.31q24.32) chr10:g.102860001_ 103480000dup	Microduplication	0.62	Likely pathogenic
II:3	seq[GRCh37]dup(10)(q24.31q24.32) chr10:g.102820001_ 103460000dup	Microduplication	0.64	Likely pathogenic

### Identification of cnvs at the single-cell level and validation using SNP based platforms

The identification of CNVs at the single-cell level and their subsequent validation using SNP-based platforms pose formidable challenges in clinical practice due to the limitations in cell acquisition arising from technical constraints. The resolution of microduplication variants at the single-cell level constitutes a pressing issue warranting attention, with the potential to enhance the realm of clinical genetic diagnosis. Consequently, to address the endeavor of identifying microduplications at the single-cell level, DNA samples were subjected to further dilution, reducing them to approximately 10 pg, corresponding to the single-cell level. This was followed by comprehensive genome amplification via MDA and MALBAC techniques, respectively. These processes facilitated the detection of copy number variations within chromosomes and mutations within genes using linkage analysis.

**Fig. 3:**
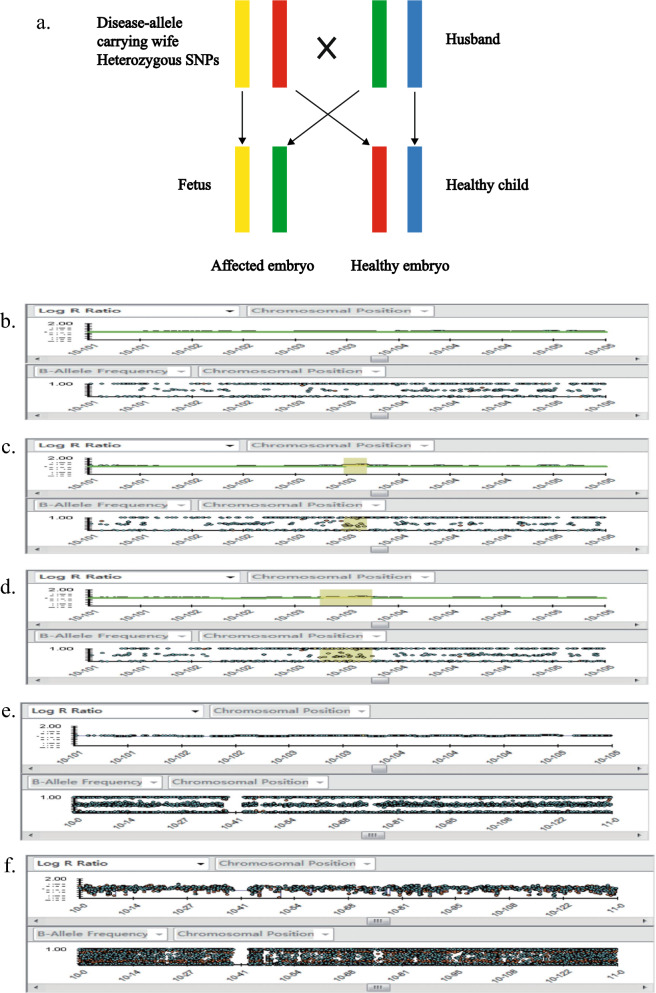
The pathogenic CNV detection on single cell level using Karyomapping. **a** Schematic representation of the linkage identified for the disease-carrying allele. Yellow indicates the allele carrying the heterozygous SNPs, which is inherited from the mother. **b**–**f**. From top to bottom represent the results of father (**b**), mother (**c**), fetus (**d**), normal son (**e**), and single cell expansion product (**f**). The selected area in the yellow box indicates the area where *BTRC* is located

Through the utilization of karyomapping technology, the process of karyotype localization was executed on couples, sons, and malformed fetuses, alongside the amplification products of individual cells. Subsequently, an elucidation of the DNA arrangement rule governing genetic material ensued. The identification of the haplotype DNA harboring pathogenic genes within this familial context was achieved by means of analysis conducted with BlueFuse Multi Software in accordance with the provided manual. As delineated in the illustrative model diagram, it was ascertained that the malformed fetus had inherited the haplotype of the mother (Fig. 3a). The presentation of the B allele Frequency plots and Log R Ratio plots adhered to stringent criteria to determine diploid presence. The yellow region denoted the locus of the *BTRC* (Fig. 3b-f). Notably, the LogR diagram exhibited an upward shift in the mother and the fetus within this familial cohort, indicative of SNP sites within this domain. Further scrutiny unveiled the presence of 230 Informative SNPs (effective SNPs) located in the 5′ terminus of the malformed fetus within the BTRC gene region, of which 53 originated from the maternal lineage and 20 from the paternal lineage. In the intragenic region, 33 active SNPs were identified, with 5 emanating from the maternal lineage and 1 from the paternal lineage. In the 3′ terminus, 287 active SNPs were observed, with 22 attributed to the maternal lineage and 45 to the paternal lineage. Subsequent analysis elucidated that the malformed fetus harbored a 442 kb copy number repeat in the 10q24.31 to 10q24.32 region. The pathogenicity of this SNP was conclusively assessed as pathogenic, including the *BTRC* gene and other genes associated with hand-foot deformity. Thus, corroborated by Karyomapping, the fetal chromosome and the genotype of the genetic disease remained entirely congruent with the SNP linkage analysis results.

### Identification of cnvs at the single-cell level and validation using next generation sequencing and single molecular sequencing methods

**Fig. 4:**
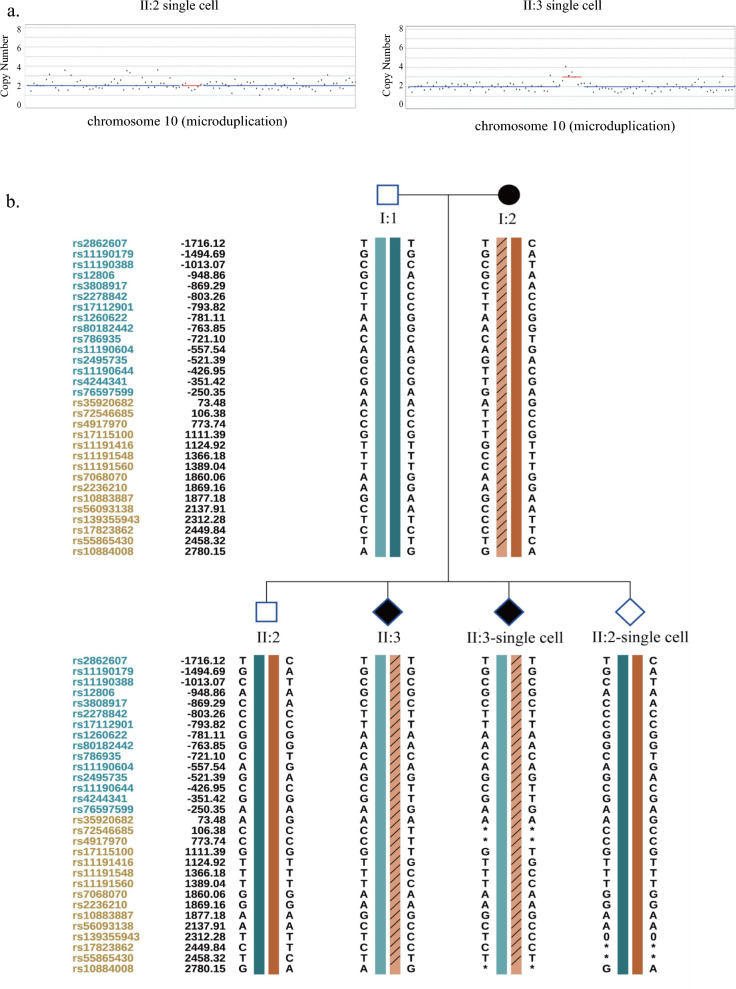

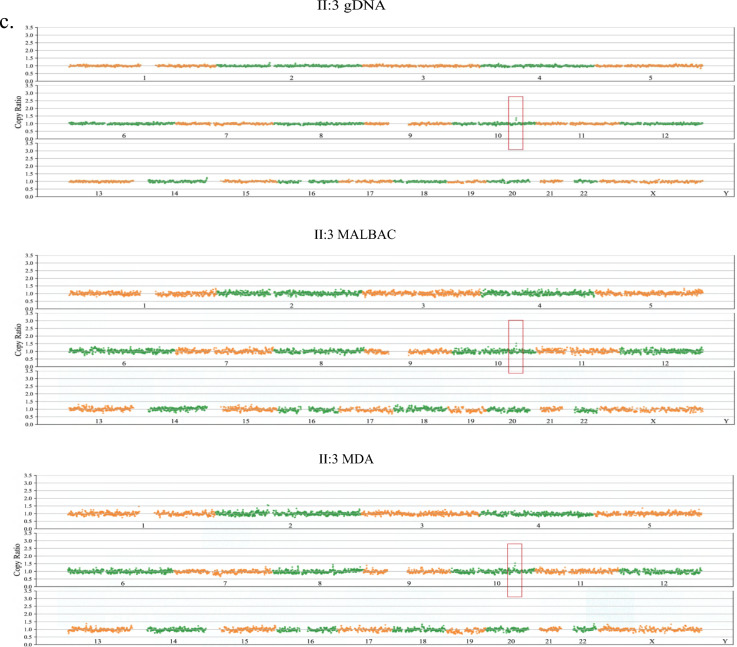
The pathogenic CNV detection on single cell level using sequencing. **a** The pathogenic CNV of the fetus at chromosome 10 using NGS. **b** The linkage analys of the family. **c** The pathogenic CNV of the fetus at chromosome 10 using single molecular sequencing method at gDNA and WGA products

Subsequently, further confirmation was undertaken through NGS analysis employing trace genomic DNA (gDNA) and the MDA products from aborted fetal tissue. The findings consistently revealed a duplication spanning 0.64 Mb. Notably, the identified gene was situated in the 10q24.31q24.32 region, and the findings disclosed the absence of the maternal mutation in the healthy son (II:2), while the aborted fetus (II:3) harbored the maternal heterozygous duplicationchr10:g.102860001_103480000dup (Fig. 4a).

To substantiate the feasibility of successfully identifying subtle variations at the single-cell level, SNP linkage analysis was conducted within this familial context. Genotypic information pertaining to SNP alleles at sites located within 2 M of upstream and downstream of the microduplication region was analyzed in the genomic DNA of the family members, as well as in the WGA products derived from single cells. As depicted in our results, the linkage analysis concurred with the findings of mutation site detection. Specifically, there were 13 informative SNPs within 1 M in healthy boy at the single-cell level (II:2-single cell), whereas the aborted fetus (II:3-single cell) exhibited 12 effective SNPs within 1 M (Fig. 4b). Based on the genotype of the SNP locus in the family member carrying the 620 kb region mutation, the haplotype carrying the 620 kb region mutation was inferred (Table 2). For instance, if rs11190644 is C/C in father, T/C in mother, and C/T in the malformed fetus, we can infer that the T bases are inherited from females, and so on for the other SNPs (we usually utilized at least five informative SNPs), then we can easily constructed the pathogenic and non-pathogenic haplotype; thus, utilizing the haplotype to deduce the inheritance of the variation. The results of SNP site analysis in single cell amplification products were congruent with those of conventional mutation detection; thus, signifying the successful recognition of 620 kb microduplications at the single-cell level in this study.

**Table 2:** Informative SNPs flanking the microduplication at chr10 of SHFM

SNP_ID	Chr	Position	Maternal hap 1	Maternal hap 2	Paternal hap1	Paternal hap2	II:2	II:2 single cell	II:3	II:3 single cell
rs2862607	chr10	99,384,124	T	C	T	T	T/C	T/C	**T**/T	**T**/T
rs11190179	chr10	99,605,556	G	A	G	G	G/A	G/A	**G**/G	**G**/G
rs11190388	chr10	100,087,170	C	T	C	C	C/T	C/T	**C**/C	**C**/C
rs12806	chr10	100,151,385	G	A	G	A	A/A	A/A	**G**/G	**G**/G
rs3808917	chr10	100,230,958	C	A	C	C	C/A	C/A	**C**/C	**C**/C
rs2278842	chr10	100,296,988	T	C	T	C	C/C	C/C	**T**/T	**T**/T
rs17112901	chr10	1,003,064,278	T	C	T	C	C/C	C/C	**T**/T	**T**/T
rs1260622	chr10	100,319,136	A	G	A	G	G/G	G/G	**A**/A	**A**/A
rs80182442	chr10	100,336,391	A	G	A	G	G/G	G/G	**A**/A	**A**/A
rs786935	chr10	100,379,146	C	T	C	C	C/T	C/T	**C**/C	**C**/C
rs11190604	chr10	102,302,457	A	G	A	A	A/G	A/G	**A**/A	**A**/A
rs2495735	chr10	102,338,609	G	A	G	G	G/A	G/A	**G**/G	**G**/G
rs11190644	chr10	102,433,046	T	C	C	C	C/C	C/C	C/T	C/T
rs4244341	chr10	102,508,577	T	G	G	G	G/G	G/G	G/T	G/T
rs76597599	chr10	100,849,895	G	A	A	A	A/A	A/A	A/G	A/G
rs35920682	chr10	101,793,725	A	G	A	A	A/G	A/G	**A**/A	**A**/A
rs72546685	chr10	103,586,376	T	C	C	C	C/C	C/C	C/T	–/–
rs4917970	chr10	104,253,735	T	C	C	C	C/C	C/C	C/T	–/–
rs17115100	chr10	104,591,393	T	G	G	G	G/G	G/G	G/T	G/T
rs11191416	chr10	104,604,916	G	T	T	T	T/T	T/T	T/G	T/G
rs11191548	chr10	104,846,178	C	T	T	T	T/T	T/T	T/C	T/C
rs11191560	chr10	104,869,038	C	T	T	T	G/G	T/T	T/C	T/C
rs7068070	chr10	105,340,059	A	G	A	G	G/G	G/G	**A**/A	**A**/A
rs2236210	chr10	105,349,161	A	G	A	G	A/A	G/G	**A**/A	**A**/A
rs10883887	chr10	105,357,181	G	A	G	A	A/A	A/A	**G**/G	**G**/G
rs56093138	chr10	105,617,908	C	A	C	A	T/T	A/A	**C**/C	**C**/C
rs139355943	chr10	105,792,280	C	T	T	T	C/T	0/0	T/C	T/C
rs17823862	chr10	105,929,840	C	T	C	C	T/C	–/–	**C**/C	**C**/C
rs55865430	chr10	105,938,324	T	C	T	T	G/A	–/–	**T**/T	**T**/T
rs10884008	chr10	106,260,146	G	A	A	G	T/T	G/A	A/G	–/–

Bold represent heterozygous SNPs inherited the mother

Concurrently, the data underwent validation utilizing the Single-Molecule Gene Sequencing approach, employing both extensive and limited DNA WGA products, which also demonstrated a 0.64 Mb duplication positioned within the 10q24.31q24.32 locus (Fig. 4c). This conveys the congruence of outcomes obtained via distinct methodologies. It is noteworthy that the MDA product’s results exhibited discretization, rendering the detection of variations challenging.

### The new strategy for identification of micro-variation

**Fig. 5:**
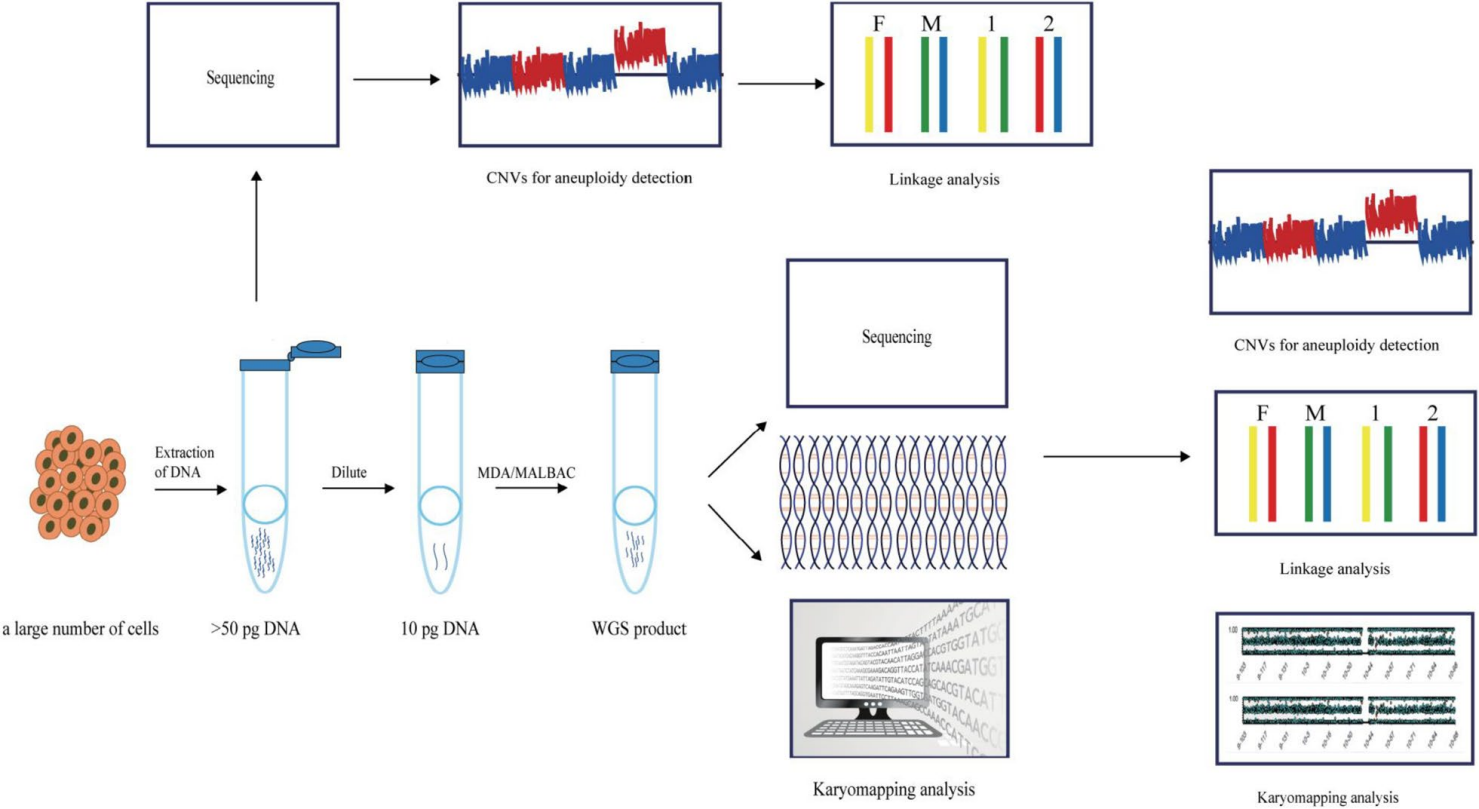
The new strategy for identification of microduplication and microdeletion at single cell level

In summary, a novel methodology for discerning microrepeat variants at the single-cell level was devised and implemented (Fig. 5). Initially, both whole blood and tissue specimens were collected from members of the SHFM3-afflicted family. Subsequently, we conducted copy number variation and single nucleotide polymorphic site detection using NGS, SNP, and single molecular sequencing at the standard DNA level. These analyses revealed a 620 kb microduplication variation in both the proband and the affected fetus. we adopted single-cell-level DNA amplification techniques employing MDA and MALBAC. Furthermore, we used single-cell level DNA for whole gene amplification by MDA and MALBAC. Additionally, Karyomapping was executed following the amplification step. Karyomapping technology employs Illumina’s SNP chip to acquire SNP site information from the target sample, which is subsequently processed using BlueFuse Multi software for comprehensive SNP analysis. Consequently, haploblock information for each embryo chromosome is obtained and visualized within the software interface. Based on the haplotype segment data, as well as the distinctions between “key SNP” and “non-key SNP” for each segment, the pathogenic gene-carrying status of each embryo chromosome was assessed. Last but not least, next generation sequencing and single molecular sequencing methods will further help us to identify microduplicate variants at the single-cell level. Our analytical findings corroborate the detection of the 620 kb micro-repeat mutation after MDA amplification at the single-cell level, with linkage analysis attributing the mutation to the maternal lineage.

## Discussion

Split-hand/foot malformation (SHFM) represents a congenital limb abnormality predominantly affecting the central rays of the autopod [15]. It can manifest either in isolation or as a component of a broader congenital anomaly syndrome. Perturbations in gene dosage due to chromosomal duplications or deletions of minute genomic segments can underlie complex clinical phenotypes. Genetic counseling and diagnostic microduplication analysis for SHFM3 patients present formidable challenges and hold paramount significance in their clinical management. Particularly pertinent is the guidance provided to patients with reproductive aspirations to prevent the transmission of this genomic variation to subsequent generations. In the present study, we successfully devised diverse methodologies to identify micro-variations, capitalizing on three distinct platforms.

For SHFM3 patients with known pathogenetic genes, the application of preimplantation or prenatal genetic diagnosis can substantially mitigate the risk of transmission to the progeny. However, conventional diagnostic methodologies entail the utilization of high-throughput sequencing to discern CNVs associated with aneuploidy, as well as SNPs for detecting single-gene point mutations. Traditional karyotype analysis, in contrast, faces challenges in detecting chromosome microdeletions and duplications below 5 Mb in size. Particularly noteworthy is the constraint imposed on the biopsy tissue employed in preimplantation diagnostic technology, as it is restricted by the presence of trace DNA. This limitation can give rise to allele deletion or loss during the process of traditional whole-genome amplification, potentially causing the misidentification of heterozygous states as homozygous. Consequently, the occurrence of false-negative or false-positive diagnostic outcomes is a distinct possibility. In our investigation, we have established a novel strategy for the identification of microduplications, and also can be applicable to microdeletions at single cell level, which can be used for embryo preimplantation genetic testing. This approach is robust for disorders like SHFM3, result in by pathogenic microduplications. Following double verification through high-throughput sequencing and SNP assays, we have successfully identified the variation locus at chr10:g.102860001_103480000dup within the family. Furthermore, we have constructed a linkage analysis diagram to scrutinize the chromosome karyotypes of the parents and their offspring.

The SNP loci within the patient’s family were subjected to in-depth scrutiny, resulting in the precise identification of mutation loci. Notably, the heterozygous mutation present in the malformed fetus was traced back to its inheritance from the mother. A pathogenic SNP was identified within the target region of chromosome 10, demonstrating an association with hand-foot division deformity. Karyomapping, in addition to providing accurate test results, exhibits commendable flexibility. Another notable advantage of Karyomapping lies in its capacity to detect chromosomal variations and ascertain their parental origins, including aneuploidy and partial chromosome deletions, all while conducting SNP genotyping. Furthermore, through the verification process involving Karyomapping and linkage analysis, we successfully circumvented the limitations imposed by alle-drop-out (ADO) stemming from WGA. ADO is critically important because it can, and not infrequently does, lead to disastrous false negative results [32]. Our achievement facilitated a more precise genetic source assessment for the family and led to the identification of a pathogenic variation in the BTRC gene within the 620 kb micro-repetition region of the family.

## Conclusions

In conclusion, our investigation confirms identification of 0.62 Mb microduplicaton at the single-cell level. This study is the first one on the development of a novel single-cell-level microduplications recognition technology, thus addressing the challenge of preventing the transmission of microduplication genetic diseases to future generations. Whether this method is feasible in other small variations needs to be further verified.

## Data Availability

The raw sequence data reported in this paper have been deposited in the Genome Sequence Archive (Genomics, Proteomics & Bioinformatics 2021) in National Genomics Data Center (Nucleic Acids Res 2022), China National Center for Bioinformation/Beijing Institute of Genomics, Chinese Academy of Sciences (GSA-Human: HRA007292) that are publicly accessible at URL: https://ngdc.cncb.ac.cn/gsa-human/s/2R6A5Qeq. The data that support the findings of this study are available from the corresponding author upon reasonable request.

## Abbreviations

SHFM: Split hand/foot malformation
WGA: Whole-genome amplification
SNP: Single nucleotide polymorphism
NGS: Next-generation sequencing
CNVs: Copy number variants
PGT: Preimplantation genetic testing
AER: Apical ectodermal ridge
PZ: Progression zone
ZPA: Polarization active zone
DSS1: Decreased sperm survival 1
DLX5: Distal-less homeobox 5
DLX6: Distal-less homeobox6
TP63: Tumor protein p63
HOXD: Homeobox
SHFLD: Split hand/foot malformation with long bone deficiency
PGT-A: Preimplantation genetic testing for aneuploidy
PGT-M: Preimplantation genetic testing for monogenic disorders
PGT-SR: Preimplantation genetic testing for structural rearrangements
ART: Assisted reproduction technology
MDA: Multiple displacement amplification
MALBAC: Multiple annealing and looping-based amplification cycles
LRR: The Log R Ratio
BAF: The B Allelic Frequency
DGV: Database of Genomic Variants
OMIM: Online Mendelian Inheritance in Man
CRL: Head and hip diameter
BPD: Double parapedal diameter
LBX1: Ladybird homeobox1
BTRC: Beta-transducin repeat containing E3 ubiquitin protein ligase
POLL: DNA polymerase lambda
DPCD: Deleted in primary ciliary dyskinesia homolog
ACMG: American Society of Medical Genetics and Genomics
LP: Likely pathogenetic
ADO: Alle-drop-out
